# Market Impacts of Reducing the Prevalence of Bovine Respiratory Disease in United States Beef Cattle Feedlots

**DOI:** 10.3389/fvets.2017.00189

**Published:** 2017-11-09

**Authors:** Kamina Keiko Johnson, Dustin L. Pendell

**Affiliations:** ^1^Animal and Plant Health Inspection Service, United States Department of Agriculture, Washington DC, CO, United States; ^2^Kansas State University, Manhattan, KS, United States

**Keywords:** bovine respiratory disease, feedlot, partial equilibrium model, reduce disease prevalence, United States

## Abstract

Bovine respiratory disease (BRD) is a common endemic disease among North American feedlot cattle. BRD can lead to significant economic losses for individual beef cattle feedlot producers through mortality and morbidity. With promising new management and technology research that could reduce BRD prevalence, this study evaluates the potential impacts of a reduction of BRD in the US beef cattle feedlot sector. Using a multi-market, multi-commodity partial equilibrium economic model of the US agricultural industry, we evaluate the market impacts of reduced BRD to producers from various livestock, meat, and feedstuffs industries. We find that as morbidity and mortality is reduced, beef cattle producers experience losses due to increased supplies (lower beef cattle prices) and increased demand for feedstuff (higher feedstuff prices). Beef cattle processors see gains as the price of beef cattle is lower, whereas feedstuff producers gain from higher feedstuff prices. Producers in the allied industries (pork, lamb, poultry, and eggs) see a small reduction in returns as consumers substitute with less expensive beef products. Consumers see gains in welfare as the increase in beef cattle supply results in lower beef prices. These lower beef prices more than offset the small increases in pork, lamb, poultry, and egg prices. Overall, the potential economic welfare change due to management and technologies that reduce BRD is a net gain for the US society as a whole.

## Introduction

Bovine respiratory disease (BRD) is endemic and one of the most common and costly diseases in commercial North American feedlots (1). The United States (US) beef industry has several endemic diseases like BRD that exhibit low mortality rates and morbidity rates with wider spread effects on production. BRD is a general term that covers upper and lower respiratory diseases in cattle caused by stress, viral infection, and/or bacterial infection (2).

The National Animal Health Monitoring System’s (NAHMS) Beef Feedlot 2011 study found that an estimated 21.2% of beef cattle (2.29 million) placed in feedlots were affected by respiratory disease (3). BRD is responsible for approximately 45–55% of all deaths in the feedlot (4). The NAHMS Beef Feedlot 2011 Study (5) reports that the direct cost of treatment of respiratory disease in feedlot cattle is USD $23.60 per case, which is nearly double the reported nominal cost in 1999 (USD $12.59 per case). The total cost for treating 2.29 million cattle for respiratory disease is, therefore, estimated to be USD $54.12 million, not including production losses due to morbidity and mortality.

## Objective

This research estimates the potential economic impact of reducing BRD in US beef cattle feedlots. This study expands on previous research by focusing on the reduction of an endemic disease rather than a foreign disease.

## Background

Over the last 33 years, a vast amount of research has focused on BRD (6). Topics range from prevention to vaccination strategies, including risk factors. BRD is a complex multi-factorial disease caused by interactions between infectious agents (e.g., viruses, bacteria, and/or parasites), environmental factors (e.g., transportation, temperature fluctuations, and ventilation), and host characteristics (e.g., immune status, genetics, and age). The clinical signs of BRD include difficulty breathing, nasal discharge, depression, fever over 40°C, and diminished or no appetite. In addition to feedlot cattle, recently weaned calves, nursing beef calves, housed dairy calves, and lactating dairy cows with lung infections that cause pneumonia are at higher risk for BRD (7).

Since stress is a factor that increases the risk of BRD, preconditioning was introduced in 1967 as a means to reduce stress (7). Central components of preconditioning include low-stress weaning, weaning several days to weeks in advance of sale, administration of clostridial vaccines, administration of vaccines for respiratory viruses and bacteria, dehorning and castration with more healing time prior to sale, and training calves to bunk feed. Other prevention methods include nutrition analysis and metaphylactic treatment.

Antimicrobials are commonly used in treating cattle with BRD. Cattle are often treated up to three times before being labeled chronically ill with the disease and thus culled from the herd or being sorted into a separate pen for feeding and monitoring.

Prevalence estimates of BRD in cattle have been a topic of research in region-specific studies (8–11) as well as for the entire United States (3, 12). The NAHMS Beef Feedlot 2011 Study reports that 97.0% of feedlots in the United States are affected by BRD (3). Within those affected feedlots, 21.2% of cattle less than 317.5 kg when placed^1^ are infected with respiratory disease and 89.6% of those cattle receive treatment. According to the NAHMS Beef Feedlot 2011 Study, the mortality rate for all feedlot cattle affected by BRD is 1.12% (3).

The NAHMS Beef Feedlot 2011 Study did not collect production performance information from cattle in the treated and recovered categories. Thus, previous literature is used here to provide estimates of the production differences between cattle never treated for BRD, treated once for BRD, and treated two or more times for BRD^2^ (13–26). All of the studies reviewed that contain a discussion on the economics of BRD cite negative impacts on common performance parameters such as feed conversion efficiency (% change in unit of feed for 0.45 kg of gain), change in time to market (days), average daily gain (kilograms gained per day), final body weight (kg), hot carcass weight (kg), marbling score, and quality grade. Cattle treated multiple times for BRD at the feedlot had reductions in average daily gain of up to 0.98 kg/day, reductions in hot carcass weight of up to 19.96 kg, and a reduction in marbling score of up to 28 points.

Additional information can be found in the reviewed articles on other potential economic consequences such as reductions in feed intake and direct cost of alternative types of treatments used. Some parameters, such as feed intake, are difficult to measure in individual cattle as they are housed and fed together in large pens. This management situation, in turn, affects reporting accuracy of certain economic parameters. Brooks et al. (13) reported a loss of USD $143.28 in net returns per head for chronic cattle, whereas Faber et al. (22) reported a loss of USD $57.48 per head. Some treatment cost estimates ranged from USD $2 per head (17) to USD $12.39 per head (22). Other studies have reported costs or lost value ranging from USD $15.57 per head to more than $151 per head (16, 23, 24, 26).

A number of studies have estimated the direct impact or direct costs of BRD, but no known study has evaluated the full market impacts that might occur if BRD were reduced. This study calculates the national market impacts—which includes mortality and morbidity resulting from BRD, competing products like pork and poultry, and input industries (feed and forage)—of reducing the prevalence of BRD.

## Methods and Data

In this study, we use a multi-market and multi-commodity quarterly partial equilibrium model of the US agricultural industry (27). The model incorporates both vertical and horizontal linkages within livestock production and through to the final consumer, as well as international trade. In addition to livestock and meat markets, grain industries are incorporated in the model because feed is a major input into livestock production. A summary of this quarterly demand and supply model is presented next [complete documentation is provided in Ref. (27)].

Final consumer demand is modeled for 10 separate goods (beef, pork, poultry, lamb, dairy, eggs, wheat, rice, coarse grains, and soy oil). Final demand (measured as per capita consumption) depends on retail price and per capita income. Demand shocks can be introduced to the model to simulate possible changes in consumer demand associated with animal disease events. Supply of final goods is modeled for the same 10 goods. Linkages between vertical segments of the livestock production industries (separate industries include beef cattle, hogs, dairy, poultry meat, layers, and sheep and lambs) occur through derived demands for animals for slaughter (net of trade), live animal inventory, and derived demands for feedstuffs. Each production sector is modeled carefully to capture dynamic inventories, including appropriate biological quarterly lags to reflect production lags, as well as linked animal flows through the various production phases. Crop supplies are modeled for wheat, coarse grains, soybeans, rice, and forage and pasture. Overall, closure of the model requires market-clearing domestic and international trade prices and quantities. Excess demand and supply equations are used to model imports and exports. Finally, vertical market prices at farm, wholesale, and retail are modeled using margin markup equations based on U.S. Department of Agriculture (USDA) margin calculations.

The structural model of demand and supply relationships is transformed through total logarithmic differentiation to enable use of pre-defined elasticities to quantify impact estimates. All economic model parameters, substitution and trade elasticities, revenue and factor shares, and livestock-feed balance information remain constant throughout the model and are defined by Paarlberg et al. (27). Updated parameters used in our model relative to the specifications of Paarlberg et al. (27) include retail-level demand elasticities for the following: beef, pork, and poultry (28); lamb (29); and milk (30). The model assumes naive price expectations for all scenarios (31).

This economic model has been applied to various species within the livestock industries, including swine (31) and poultry (32–34). Previous research has used this and related models to estimate the market impacts of a negative exogenous shock to the supply of beef cattle in the United States due to a disease outbreak (27, 35–41). One study by Seitzinger et al. (42) evaluated the economic impact of eradicating three endemic ovine diseases in the United States. Our research is the first to model a positive exogenous supply shock to the beef industry, specifically an increase in beef production as a result of a reduction in BRD. Another feature of this study is the introduction of a shock to feed consumption, which represents increased demand for feed as morbidity declines due to the reduction of BRD.

**Figure 1:**
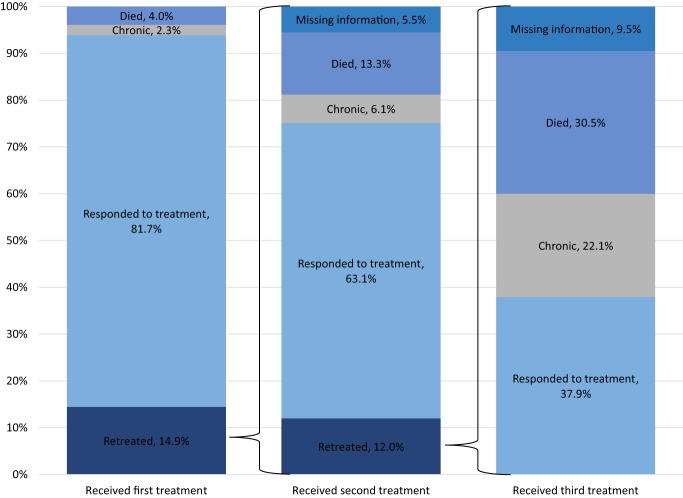
US feedlot cattle affected by bovine respiratory disease, measured as percentage of national herd by category. Source: National Animal Health Monitoring System Beef Feedlot 2011 Study.

Our study uses feedlot cattle inventory data collected by USDA-National Agricultural Statistical Service (NASS) (43) and the current estimated levels of mortality (1.12%) and morbidity (16.58%) from the NAHMS Study as a baseline for BRD disease prevalence. Figure 1 outlines data from the NAHMS Study showing the percentage of cattle affected with BRD in US beef cattle feedlots in 2011 for the following categories of affected and treated cattle: died, diagnosed as chronic, responded to treatment, retreated, cattle with missing information, and number of treatments. Among the 89.6% of affected cattle that receive a first treatment for BRD, 14.9% also receive a second treatment, and then 12% of those cattle receive a third treatment for BRD. Interestingly, Figure 1 reveals evidence to support the current production management decision to treat cattle up to three times for BRD. Of the cattle that receive a third treatment for BRD, 37.9% respond favorably to the treatment, 22.1% are diagnosed chronically ill, and 30.5% die despite the treatment. When comparing the percentage of inventory that responds to the treatment and the percentage of inventory that dies for each treatment round, industry makes the decision to stop treating cattle as the percentage that respond to treatment and percentage that die from BRD approach a similar amount of cattle (37.9 and 30.5%). Feed ratio parameters in the partial equilibrium economic model are 50% feed grains, 1.6% soybean meal, 1.4% wheat, and 47% forage. The changes in feed consumption vary by each category of feedlot cattle not affected by BRD in the BRD reduction scenario (Table 1) with those cattle not treated and recovered requiring less feed as they become more feed efficient and cattle diagnosed as chronic requiring more feed (13–26).

**Table 1:** Feed consumption level and changes in feed consumption for cattle affected under the BRD baseline scenario but not affected under the BRD reduction scenario, per-head, by category of illness.

Categories of illness under baseline scenario	Feed consumption level under BRD baseline scenario, per-head (kg)	Feed consumption level under BRD reduction scenario, per-head (kg)	Change in feed consumed under BRD reduction scenario, per-head (kg)
Cattle not affected by BRD	1,421.30	1,421.30	0.00
Cattle affected by BRD and treated once and recovered	1,527.46	1,421.30	−106.14
Cattle affected by BRD and treated twice and recovered	1,585.37	1,421.30	−164.20
Cattle affected by BRD and treated three times and recovered	1,643.27	1,421.30	−221.81
Cattle affected by BRD and diagnosed as chronic	310.91	1,421.30	1,106.76

*Sources: Literature*.

*BRD, bovine respiratory disease*.

## Scenarios

The model described above includes the baseline BRD prevalence and is referred to as the BRD baseline scenario. We compare the BRD baseline scenario to a BRD reduction scenario defined as a linear reduction of BRD prevalence, resulting in a 50% reduction at the end of 3 years (12 quarters). Hypothetical programs capable of reducing BRD could involve treatment plans, selection criteria models, the development of new vaccines and protocols, and genetic selection, among others. Management tools that incorporate historical and risk factor information (e.g., history of source herd) could be used to assist in the decision-making process when purchasing cattle or managing purchased cattle to reduce BRD. Some research has identified risk factors for developing BRD (44–46), which could inform selection or monitoring strategies (47). These could, in turn, decrease the risk of developing BRD or improve the detection time. Predictive models may prove very useful in managing BRD in feedlot cattle (48) as would technology to improve case definitions, diagnosis, and targeted antibiotic use (49). Production management practices, such as observing feeding behavior, could also be used to predict the hazard of BRD before clinical signs appear and thus accelerate treatment times (50). Wider adoption of more effective vaccination protocols (e.g., new vaccine, vaccination administration timing, animal age, and vaccination location site) can provide additional protections against BRD (51). Research in genomics to identify and select for cattle less susceptible for BRD is another promising area (52, 53).

## Exogenous Shocks

Using the USDA-NASS cattle on feed inventory from 2011 and the percentage of affected cattle shown in Figure 1, the percentage of cattle on feed inventory is calculated for all cattle categories in the BRD baseline scenario (Table 2). The percentage of cattle on feed for all categories in 2011 serves as the baseline BRD prevalence level and is assumed to be the same through the following years. The BRD reduction scenario has a target reduction of 50% prevalence (21.2–10.6%) and starts in Quarter 1 2015 and ends in Quarter 4 2017.

**Table 2:** Number of cattle on feed and percentage of cattle on feed inventory affected by BRD, by category of cattle and scenario.

Scenario/year	BRD baseline scenario 2011	BRD baseline scenario 2011	BRD reduction scenario Quarter 4 2017

Category of cattle	Percentage of inventory^a^	Number of head^b^	Percentage of inventory^a^
Cattle on feed in affected feedlots	100.00	11,703,050	100.00
Cattle not affected by BRD	78.80	9,222,003	89.40
Cattle affected by BRD that did not receive first treatment	2.20	258,029	1.10
Cattle affected by BRD that received first treatment	19.00	2,223,580	9.50
Cattle affected by BRD that responded to first treatment	15.52	1,816,664	7.76
Cattle affected by BRD that received first treatment but were diagnosed as chronic^c^	0.44	51,142	0.22
Cattle affected by BRD that received first treatment but died	0.76	88,943	0.38
Cattle affected by BRD that received first treatment and second treatment	2.83	331,313	1.42
Cattle affected by BRD that responded to second treatment	1.79	209,059	0.89
Cattle affected by BRD that received second treatment but were diagnosed as chronic	0.17	20,210	0.09
Cattle affected by BRD that received second treatment but died	0.38	44,065	0.19
Cattle affected by BRD that received second treatment but no information is available	0.16	18,222	0.08
Cattle affected by BRD that received third treatment	0.34	39,758	0.17
Cattle affected by BRD that responded to third treatment	0.13	15,068	0.06
Cattle affected by BRD that received third treatment but were diagnosed as chronic	0.08	8,786	0.04
Cattle affected by BRD that received third treatment but died	0.10	12,126	0.05
Cattle affected by BRD that received third treatment but no information is available	0.03	3,777	0.02

*^a^May not add to 100% due to multiple responses or unspecified*.

*^b^May not add to total number of head due to percentage not adding to 100*.

*^c^Cattle shipped for slaughter prior to reaching normal slaughter weight*.

*Sources: NASS, NAHMS Beef Feedlot 2011 Study, and Paarlberg et al. (27)*.

*BRD, bovine respiratory disease; NAHMS, National Animal Health Monitoring System; NASS, National Agricultural Statistical Service*.

The BRD reduction scenario uses a linear reduction of BRD prevalence across that time period, which translates to a 4.17% prevalence reduction each quarter for 12 quarters. The linear reduction in the prevalence of BRD by 50% is achieved over 12 quarters (3 years) and maintained for 4 additional quarters (1 year). The annual cattle inventory that were affected by BRD in the BRD baseline scenario that are not affected in the BRD reduction scenario are equally dispersed across the four quarters of each year. The percentages of cattle on feed inventory for all cattle categories in the BRD reduction scenario for Quarter 4 of 2017 are also presented in Table 2.^3^

As a result of this hypothetical reduction of BRD prevalence, all categories of affected cattle have been reduced by 50% in the BRD reduction scenario. For comparison, the percentage of cattle on feed affected by BRD in the BRD reduction scenario is 10.6%, which is half of the amount in the BRD baseline scenario, 21.2%. The percentage of cattle not affected by BRD has increased from 78.8% to 89.4% to represent the additional proportion of cattle not affected by BRD in the BRD reduction scenario that were affected in the BRD baseline scenario. Consequently, by Quarter 4 2017 half of the cattle that were treated for BRD, diagnosed as chronic with BRD, or died from BRD in the BRD baseline scenario are not affected by BRD in the BRD reduction scenario. Essentially, the number of feedlot cattle and beef supplied to the market increases slowly each quarter as less feedlot cattle are affected by BRD.

Additionally, feedlot cattle that are treated for BRD and recovered have a lower feed efficiency compared to cattle not affected by BRD. For each quarter in the BRD reduction scenario there are fewer feedlot cattle treated for BRD, and since those cattle are now more feed efficient they require less feed to reach market weight and will go to market earlier^4^ than they otherwise would. Feedlot cattle diagnosed as chronic with BRD and shipped to slaughter prior to reaching market weight consume less feed compared to cattle not affected by BRD. For each quarter in the BRD reduction scenario there are fewer feedlot cattle diagnosed as chronic with BRD, and these cattle will consume more feed and go to market at a later date.

Since potential BRD reduction strategies are still under development, it is difficult to estimate their cost of implementation at the producer level, without the adoption rate and the cost of such a program on a national scale. In the absence of these costs, our research assumes that implementing such a strategy is cost-effective. This requires the marginal cost of a strategy to be less than or equal to the marginal benefit gained from it. The marginal benefit gained includes avoided costs or losses relative to current practices and decisions. Using these parameters and assumptions, the net production shock of reducing BRD prevalence is a positive shift in the supply curve (or increase in supply) for beef feedlot cattle in our model (Table 3).

**Table 3:** Bovine respiratory disease reduction exogenous shocks imposed on the model, by quarter.

Quarter	Change in slaughter (%)	Change in feed grain use (%)	Change in soybean meal use (%)	Change in wheat use (%)	Change in forage use (%)
2015-Q1	0.155	0.001	0.000	0.001	0.000
2015-Q2	0.309	0.003	0.000	0.002	0.001
2015-Q3	0.464	0.007	0.001	0.002	0.001
2015-Q4	0.618	0.002	0.001	0.003	0.003
2016-Q1	0.773	0.004	0.001	0.004	0.002
2016-Q2	0.927	0.008	0.001	0.005	0.002
2016-Q3	1.082	0.016	0.001	0.005	0.002
2016-Q4	1.236	0.005	0.001	0.007	0.006
2017-Q1	1.391	0.007	0.001	0.007	0.004
2017-Q2	1.545	0.013	0.002	0.008	0.003
2017-Q3	1.700	0.026	0.002	0.008	0.003
2017-Q4	1.854	0.007	0.002	0.010	0.008
2018-Q1	1.854	0.010	0.002	0.010	0.005
2018-Q2	1.854	0.016	0.002	0.009	0.003
2018-Q3	1.854	0.028	0.002	0.009	0.004
2018-Q4	1.854	0.007	0.002	0.010	0.008

Due to the increase in beef cattle supply (increase in feedlot cattle not affected by BRD under the BRD reduction scenario), there is a subsequent increase in demand for feedstuffs, specifically, feed grains, wheat, soybean meal, and forage. The percentage of affected cattle in Table 2 and the feed consumption changes in Table 1 are used to calculate the change in feed demand by category. The net feed consumption shock of reducing BRD prevalence for all categories of affected cattle is a small positive shift in the demand curve (or increase in demand) for feed for feedlot cattle in our model (Table 3). Feed consumption changes for cattle that died from BRD under the baseline scenario but now survive in the BRD reduction scenario are already incorporated into the model on the supply side; thus, there is no additional exogenous shock for feed consumption changes for these cattle.

The net exogenous production and feed consumption shocks in Table 3 are a percentage change in beef production or feed consumption and introduced into the partial equilibrium economic model during Quarter 1 of 2015 through Quarter 4 of 2018. To provide more clarification on how these numbers are calculated we present an example for the Quarter 1 2015 change in slaughter. A reduction in BRD prevalence of 4.17% equates to 94,419 fewer cattle treated and recovered, diagnosed as chronic, or dying. This results in a net 0.155% increase in cattle supplied when considering the cattle not affected in the BRD reduction scenario that were affected in the baseline scenario. In addition, these cattle are eating more (dead and chronic) or less (treated and recovered) feed, with a small net positive shock in feed consumption of up to 0.001% increase for the four feedstuff types. Also, notice how the change in slaughter slowly increases throughout time, starting at 0.155% and ending at 1.854% by Quarter 4 2017. This represents the increase of beef supplied to the market from the cattle that were affected by BRD in the baseline scenario that are not affected in the reduction scenario as the prevalence decreases linearly to a 50% reduction by Quarter 4 2017 and remains constant through 2018. The percentage change in feed stuff fluctuates across time as the increased demand in feed is compared to the feed supply values in the model.

Finally, we assume that domestic and international consumers would not change their consumption patterns of beef products due to the reduction in BRD prevalence. Thus, shocks to domestic demand and international trade were not incorporated into our model.

The shocks from Table 3 are added to the base demand and supply amounts in the appropriate equations for each commodity affected in each quarter. The model then resolves for market-clearing quantities and prices using the new demand or supply level for each quarter. The calculated outputs of the model highlighted in this research are the changes in returns to capital and management and the consumer welfare from reductions in BRD prevalence. The returns to capital and management are the margins producers are earning, whereas economic welfare of consumers is measured by the consumer surplus, which is the difference between what consumers are willing to pay and what they must pay for each unit consumed.

## Results

The change in returns to capital and management of producers and change in consumer welfare are presented in Table 4. These changes reflect the differences in margins producers receive and consumer welfare under the BRD reduction scenario. Across the 16 quarters, beef cattle producers (beef cow-calf and feedlot producers) lose USD $4,965 million due to lower beef cattle prices and higher feedstuff costs. This dollar value does not account for the direct cost savings related to treating fewer cattle. However, the $4,965 million loss to beef cattle producers does include the following from the cattle that are not affected by BRD in the reduction scenario, but were affected in the BRD baseline scenario:

- cost savings from improved feed efficiency from cattle that are not being treated,
- accelerated revenue generation from cattle that are not being treated, and thus arrive at market sooner,
- increased cost of feed from cattle that are not diagnosed as chronic with BRD or dying that are staying in feedlots longer to reach full market weight,
- increased beef revenue from cattle that are not diagnosed as chronic with BRD arriving at market at a heavier weight,
- increased beef revenue from cattle that are not dying from BRD, and
- all the market price reactions to an increased demand on feedstuff and increased supply of beef.

**Table 4:** Economic impacts for change in returns to capital and management and consumer welfare resulting from reduction in BRD prevalence.

	Millions USD $				
Industry/sectors	2015	2016	2017	2018	Total
Beef processors	$122.57	$381.67	$684.40	$872.45	$2,061.09
Beef cattle producers	−$300.57	−$925.13	−$1,644.52	−$2,094.48	−$4,964.70
Pork processors	−$0.13	−$0.40	−$0.60	−$0.61	−$1.73
Pork producers	−$0.74	−$2.63	−$4.84	−$6.53	−$14.73
Lamb processors	$0.00	$0.00	$0.00	$0.00	−$0.01
Lamb and sheep producers	−−$0.04	−$0.15	−$0.29	−$0.43	−$0.90
Chicken meat	−$0.96	−$3.57	−$6.23	−$8.13	−$18.89
Turkey meat	−$0.32	−$0.91	−$1.57	−$1.87	−$4.66
Eggs	−$0.10	−$0.74	−$1.49	−$2.57	−$4.89
Milk and dairy	$6.88	$26.63	$48.82	$64.54	$146.87
Forage	$23.91	$67.95	$129.76	$186.59	$408.20
Coarse grains	$2.82	$10.10	$21.82	$40.01	$74.75
Soybean processing	$0.10	$0.43	$0.84	$1.64	$3.01
Wheat	$0.31	$0.81	$2.03	$3.09	$6.24
Rice	$0.01	$0.06	$0.19	$0.43	$0.69

Total change in returns to capital and management	−$146.24	−$445.89	−$771.67	−$945.86	−$2,309.66
Consumer welfare	$277.87	$809.04	$1,397.75	$1,705.73	$4,190.39

Over the same 16 quarters, beef processors gain USD $2,061 million due to lower beef cattle prices that result from an increased supply of cattle due to the decrease in BRD prevalence. Additionally, grain and feedstuff producers benefit by USD $493 million over 16 quarters due to increased demand for feedstuffs.

Because feedstuff prices increase, dairy producers reduce dairy production resulting in increased milk prices. The increase in milk prices offset the increase in feedstuff prices resulting in a very small (0.02–0.48%) increase in returns to capital and management for dairy producers. Pork, lamb, and poultry producers all experience small losses due to increased feedstuff costs and downward pressure on live animal prices from the retail level. Pork, lamb, and poultry meat prices, as well as egg prices, decrease over the 16 quarters due to consumers substituting away from these products to beef as beef becomes relatively less expensive.

The net impact to the beef cattle industry (returns to cattle producers and beef processors) over 16 quarters is a loss of USD $2,904 million, driven predominantly by the losses of beef cattle producers. The net impact to all other industries, excluding the beef cattle industry, is a gain of USD $594 million. The total change in returns from capital and management to all producers is a loss of USD $2,310 million, whereas consumers gain USD $4,190 million in welfare across the 16 quarters, driven mainly by reduced beef prices.

Beyond 16 quarters we assume that the disease prevalence reaches a new steady state, such that the positive exogenous shocks used in the model will no longer be necessary, as they become a part of the new baseline (i.e., the exogenous supply shocks become zero). When the exogenous shocks become zero, the losses to the beef cattle industry, the gains to grains and feedstuffs, and gains to consumers will eventually reach a new long-run equilibrium.

## Discussion

Bovine respiratory disease is an endemic disease and one of the most common and costly diseases in commercial North American feedlots (1). Previous estimates of the annual economic losses from death, reduced feed efficiency, and treatment cost of BRD have ranged from USD $800 to $900 million (54). Producers are currently using antibiotics to treat their cattle with BRD in hopes they will recover. Advances to improve those treatments may be met with the challenge of needing to reduce antimicrobials on livestock operations. New management tools and technologies that may reduce the prevalence of BRD are currently being discussed and explored. These options would have varied adoption rates, levels of effectiveness, time to reduce BRD prevalence, implementation costs, and aggregated benefits. Any BRD reduction program with widespread adoption in the beef cattle feedlots would result in an increase in beef produced in the United States with additional impacts on the allied industries. Therefore, better understanding of the magnitude and scope of the impacts to the beef and allied industries is needed to assist with decision making.

Using a multi-market, multi-commodity partial equilibrium model, this analysis demonstrates the possible market impacts of reducing BRD prevalence. Reducing BRD prevalence would result in increased supplies of beef cattle through lower morbidity and mortality rates. The increase in beef cattle supplies would result in an increased demand (higher prices) for feedstuffs and declines in demand (lower prices) for competing protein sources. The net effects are losses to the beef cattle industry (beef cattle producers and beef processors), positive and negative changes in returns to capital, and management for feedstuffs and allied livestock industries, respectively. A positive net impact is felt by consumers due to lower beef prices. An overall positive net societal impact would be experienced with a 50% reduction of BRD. This research highlights the tradeoffs that occur when reducing the prevalence of an endemic, low mortality disease that has negative production impacts. This information can be used to support animal health decisions made at an aggregated level, encouraging a continued focus on diseases that have a high risk of large economic losses.

Although there is a net gain between producer return to capital and management and consumer welfare changes with a reduction in BRD prevalence, individual feedlot producers are likely to be the decision makers on adopting a program that would reduce BRD prevalence in their herds. When considering the budget of a single operation, but ignoring potential market price impacts, those producers with cattle affected by BRD appear to be able to spend up to USD $23.60 per head on a program to avoid BRD and still break even (using NAHMS Beef Feedlot 2011 Study data). Spending less than USD $23.60 per head on a program to avoid BRD would appear to result in a net savings. However, this research shows that lower beef cattle prices and higher feedstuffs costs result in decreases in capital and management, and those losses will need to be considered when investing in a new prevention program. Likely, producers with BRD in their feedlots that are also early adopters may see a net gain, but as adoption rates increase the beef supplies will increase, which increases feed prices and decreases the price received for beef. It is possible that late adopters will not realize these gains (or possibly a net loss) for their efforts to reduce BRD prevalence in their feedlot. An area of future research would be investigating what types of technology and treatments should be explored, as well as measuring the true magnitude and speed of BRD reduction when these are adopted. Additional research could evaluate the economic impacts of new BRD reduction programs at the farm level and the subsequent impacts downstream.

## USDA EEO Statement

The U.S. Department of Agriculture (USDA) prohibits discrimination in all its programs and activities on the basis of race, color, national origin, age, disability, and where applicable, sex, marital status, familial status, parental status, religion, sexual orientation, genetic information, political beliefs, reprisal, or because all or part of an individual’s income is derived from any public assistance program. (Not all prohibited bases apply to all programs.) Persons with disabilities who require alternative means for communication of program information (Braille, large print, audiotape, etc.) should contact USDA’s TARGET Center at 202.720.2600 (voice and TDD). To file a complaint of discrimination, write to USDA, Director, Office of Civil Rights, 1400 Independence Avenue, SW, Washington, DC 20250-9410, or call 800.795.3272 (voice) or 202.720.6382 (TDD). USDA is an equal opportunity provider and employer.

## Author Contributions

KJ and DP have made substantial, direct, and intellectual contribution to the work and approved it for publication.

## Conflict of Interest Statement

The authors declare that the research was conducted in the absence of any commercial or financial relationships that could be construed as a potential conflict of interest.

## Footnotes

^1^The estimates for the cattle less than 317.5 kg were used instead of the all cattle or cattle over 317.5 kg estimates, because the cattle less than 317.5 kg estimates were higher and we knew that the beef production and feed shocks were going to be even smaller if we used lower percentages of cattle being affected.

^2^The “never treated” group could include cattle that remained healthy in the feedlot and cattle that contracted BRD, but were never treated. Therefore, it is difficult to compare these three groups and make conclusions based on economic differences.

^3^Due to multiple responses or unspecified data the percentage of inventory does not add up to 100% for each category of treated cattle. For the second and third treatments, this results in missing information for a small amount of cattle. Mathematically, these animals are still included in the model under the responded to treatment categories as the status of those cattle that did not not respond is still tracked to the next level of treatment.

^4^The cattle affected by BRD that responded to the first treatment go to market 13 days earlier, whereas cattle that responded to the second treatment go to market 21 days earlier, and cattle that responded to the third treatment go to market 29 days earlier.

## Abbreviations

APHIS, Animal and Plant Health Inspection Service; BRD, bovine respiratory disease; NAHMS, National Animal Health Monitoring System; NASS, National Agricultural Statistical Service; US, United States; USDA, United States Department of Agriculture; VS, veterinary service.
